# L’espace vécu and Its Perturbations in Schizophrenia: Systematic Review and Meta-analysis of Altered Body-Centric Metrics—Personal and Peripersonal Space

**DOI:** 10.1093/schbul/sbae159

**Published:** 2024-10-03

**Authors:** Andrea Raballo, Michele Poletti, Francesco Bevione, Maria Carla Lacidogna, Antonio Preti

**Affiliations:** Chair of Psychiatry, Faculty of Biomedical Sciences, University of Southern Switzerland, Lugano, Switzerland; Cantonal Sociopsychiatric Organisation, Mendrisio, Switzerland; Department of Mental Health and Pathological Addiction, Child and Adolescent Neuropsychiatry Service, Azienda USL-IRCCS di Reggio Emilia, Reggio Emilia, Italy; Department of Neuroscience, University of Turin, Turin, Italy; Department of Neuroscience, University of Turin, Turin, Italy; Department of Neuroscience, University of Turin, Turin, Italy

**Keywords:** personal space, peripersonal space, interpersonal distance, espace vécu, schizophrenia, psychosis

## Abstract

Subtle distortions of the experience of lived space have long been associated with schizophrenia. Although a body-centric transformation of space is considered an essential component of anomalous subjective experience in schizophrenia, its impact on the 2 major body-centric spatial constructs, that is, personal space (PS) and peripersonal space (PPS), is still not clear. This systematic review and meta-analysis have been set up to: (1) summarize the evidence on putative extensional differences of PS and PPS in schizophrenia as compared with controls, and (2) evaluate the quality and the limitations of available studies on the topic. Four electronic literature databases (MEDLINE, EMBASE, PsychINFO, and CINAHL) were searched with the keywords “Personal space OR Interpersonal distance AND Schizophrenia,” “Peripersonal space AND Schizophrenia” from inception until December 31, 2023, resulting in 15 studies on PS and 5 studies on PPS included in this systematic review. The 12 studies on PS included in the meta-analysis revealed that individuals with a diagnosis of schizophrenia place a larger interpersonal distance from the stimuli than controls, with a moderate effect size in both the fixed-effect model (Hedges’ g = 0.558 [95% confidence interval, CI: 0.445–0.671]; *z* = 9.67; *P* < 0.0001) and the random effects model (0.547 [0.294–0.799]; *z* = 4.77; *P* = 0.0006). The 5 studies included in the meta-analysis on PPS showed that individuals with a diagnosis of schizophrenia exhibit a narrower PPS than the controls at the fixed-effect (Hedges’ g = 1.043 [95%CI: .739–1.348]; *z* = 6.72; *P* < .0001), but not at the random effects model (1.318 [−0.721 to 3.359]; *z* = 1.79; *P* = .147). Heterogeneity was substantial in both meta-analyses. Overall, the findings indicate that both body-centered space constructs (PS and PPS) are affected in schizophrenia, with an enlargement PS and a reduction PPS, thereby supporting the distinction of these constructs. These modifications cohere with the subjective transformation of the lived space (aka espace vécu) reported in classical psychopathology and may be promising, neurodevelopmentally grounded, biomarkers of vulnerability to schizophrenia and its spectrum conditions.

*“It can be said that if we share a world in common, it’s because we share a body in common. If the actions of others appear meaningful and understandable to me, it’s because they stem from a body similar to mine”*

(Stanghellini, Mancini, 2018, 97)

*“There is a distance which separates me from life or, rather, which unites me with life. There is always a free space in front of me in which my activity can develop”*

(Minkowski 1970, 403).

*“The experiential structure is transformed in such a way that each aspect of the patient’s perceptual field is related back to him…”*

(Conrad 1959, 405)

## Introduction

Subtle distortions of the experience of lived space (aka *espace vécu*) have long been associated with schizophrenia spectrum disorders (SSD), their prodromal states, and schizotaxic or family high-risk conditions.^1–13^ As explicitly thematized by Jaspers,^1^ Minkowski,^2^ and Conrad^4^ among other classical authors and found in first personal autobiographical narratives,^14,15^ patients with vulnerability to SSD manifest salient changes in the experiential structure of lived space, for example, self-reference, which are central to the elaboration of psychotic experiences since their very first incubation.

Indeed, anomalous subjective experiences of spatiality are enlisted in phenomenological semistructured interviews as the Bonn Scale for the Assessment of Basic Symptoms,^16^ the Examination of Anomalous Self Experiences,^17^ the Schizophrenia Proneness Inventory,^18^ and the Examination of Anomalous World Experience (EAWE).^19,20^

### The Embodied Self and the espace vécu in Schizophrenia

According to phenomenology, the pre-reflective (Basic or Minimal) Self is rooted in the multisensory body.^21^ The unique characteristics of the Basic Self, including the sense of agency, body ownership, and the distinction between oneself and others, are believed to develop gradually during infancy. This development occurs through the repetitive and consistent engagement in sensorimotor actions within the surrounding environment, coupled with contingent proprioceptive signals.^22–24^

In the context of schizophrenia, neurodevelopmental constraints may hinder the integration of external, internal, and proprioceptive signals, disrupting the development of the Basic Self. This disruption, in turn, appears to contribute to a diminished implicit attunement between the Self and the body, leading to a sense of disembodiment and interfering with the boundaries between the Self and others.^25–30^ Self-disorders related to an altered embodiment emerge early and specifically aggregate in SSD,^31,32^ encompassing a pathological detachment from the bodily side of the Self along 2 extremes, which have been captured by Stanghellini as “deanimated body” (ie, a body deprived of the possibility of living personal experience as its own) or “disembodied spirit” (ie, a sort of abstract entity that contemplates its own existence from outside, in a third-perspective rather than in a first-perspective).^21^ Self-disorders may also result in difficulties interacting with the environment in terms of perturbations of the *espace vécu.*^7^ In normal circumstances, the lived space streams as “*not homogeneous, but centered on the person and his body, characterized by qualities such as vicinity or distance, wideness or narrowness, connection or separation, attainability or unattainability*.”^33^

Instead, in prodromal or earlier clinical stages of schizophrenia, the *espace vécu* may be permeated by a sense of centrality, that is, an abnormal feeling of being a focal point at the center of the world^4,5,12,13^ or by an abnormal blurriness and fragmentation of the spatial boundaries between self, body, and world.^34^ Alterations of spatiality may persist also in more advanced stages and also emerging clinical symptoms of schizophrenia involve the body-centric space. For example, patients may perceive being invaded by hallucinatory intruders or delusional persecutory agents^35,36^ and may feel passive external influences crossing and disregarding their space and gradually acclimate to an expanded space, maintained by bizarre or peculiar behaviors.^37,38^

## Aims of the Study

However, while this vast and stratified array of psychopathological research points to a transformation of the subjective experience of spatiality (eg, self-reference, derealization, shift in embodied first-personal perspective) as an essential component of vulnerability to SSD, its impact on the 2 major body-centric spatial constructs implicated in schizophrenia (ie, personal space (PS) and peripersonal space (PPS), Box 1) is still not clear. PS refers to the physical zone around an individual that they consider as their private area, experiencing discomfort when others intrude. PPS is the area within arm’s reach used for object interactions. Thus, PS deals with social and emotional boundaries, whereas PPS is involved with spatial interactions with objects.

**Box 1.** Personal Space and Peripersonal Space: Overlap and Differences

	Personal Space (PS)	Peripersonal Space (PPS)
Definitions	PS is a psycho-sociological concept which captures a sphere an individual considers theirs to live in. That is, an area individuals maintain around themselves into which others cannot intrude without arousing discomfort or even withdrawal.^39–44^	PPS encompasses the space immediately surrounding the body as a sphere of action within reaching distance and is mostly characterized as a neurocognitive construct indexing the “plastic, pragmatic and goal-directed multisensory buffer that connects the brain-body with its immediate environment.”^45–48^
Key features	Captures more extensively an intersubjective interface	Encompasses a more motor-oriented sphere; describes the region near the body where physical interactions with objects occur
Assessment	The most used is the*Stop Distance paradigm*In this task a participant faces another person walking toward her/ him (the confederate)*Passive version*: participants stay still and have to stop the approaching confederate at the latest separating distance they feel comfortable with. *Active version*: roles are reversed and the confederate stays still while participants move towards him/her to stop at a comfortable separating distance	Multiple experimental procedures have been proposed for PPS^47,48^For example, participants are asked to respond as fast as possible to a tactile stimulus administered on their hand, while task-irrelevant sounds were presented, giving the impression of a sound source either approaching toward their bodies or being static. Tactile stimuli either preceded the sounds or were given at 5 different temporal delays from sound onset, corresponding to 5 possible distances from the participants. It has been shown that close (ie, within PPS), but not far, sounds boost tactile reaction times (RTs). Hence, looming sounds allowed measuring the boundary of the participant’s PPS, as the distance where sounds affected tactile RTs.
Experimental findings	PS size and responses to PS violations can vary depending on cultural, social, and situational factors, as well as personal preferences.^41,44^	Modulated by tool use^47–49^

Therefore this systematic review and meta-analysis have been set up to:

1. summarize the evidence on the modification of the PS and of PPS in schizophrenia compared to controls and
2. evaluate the quality and the limits of the studies on the topic.

## Methods

The reporting of this systematic review follows the indications of the most recent versions of the Preferred Reporting Items for Systematic Reviews and Meta-Analyses (PRISMA).^50,51^

### Eligibility Criteria

Inclusion criteria were: published studies including patients diagnosed with schizophrenia who were compared with controls, reporting measures of PS or PPS; detailing the numerical results of the analysis. Exclusion criteria: studies published in abstracts or thesis; reporting data on healthy subjects measured with a self-report questionnaire. We did not look for grey literature since there is evidence that selection bias in unpublished literature is higher than in published literature.^52,53^

### Search Strategy

Four electronic literature databases were searched: PubMed/MEDLINE, Excerpta Medica dataBASE (EMBASE), PsycINFO, and Cumulative Index to Nursing and Allied Health Literature (CINAHL). This combination of platforms is probably to produce the best unique references.^54^

The following keywords were applied: “Personal space OR Interpersonal distance AND Schizophrenia” and “Peri personal space AND Schizophrenia.” On each database, the search was from inception until December 12, 2023, and it was conducted on January 13, 2024.

Each platform was searched for individually. No language or other restrictions were applied to any of the searches. The reference lists of included studies were also manually searched.

### Data Extraction and Assessment of Methodological Quality

Four authors (F.B., M.C.L., M.P., and A.P.) independently screened articles’ titles, abstracts, and full text and extracted data. The following information was extracted from each article: location of the study; criteria for diagnosis of schizophrenia; sample size of the groups; proportion of men in the sample, mean age of the participants; nature, characteristics, and metrics of the measures used to score PS or PPS; scores of the participants on the measure of PS and PPS.

Critical appraisal of the quality of included studies was carried out independently by 2 reviewers (M.P. and A.P.) with the Newcastle-Ottawa Scale for assessing the quality of nonrandomized studies in meta-analyses.^55^

Any differences in assessment results between reviewers were resolved to consensus with an experienced reviewer (A.R.). Adherence to the proposed criteria was classified as “low risk of bias,” and lack of adherence was classified as “high risk of bias.” According to the met criteria, the study was further categorized as “good” or at low risk of bias, “fair” or with some concerns of bias, or “poor” or at high risk of bias.

### Data Synthesis

Meta-analysis was done with the following packages running in R version 4.2.2: “meta,” “metafor,” and “MAd.”^56–59^ Threshold for statistically significant results was set at *P* < .05, with both interval of 95% CI above or below the unit (depending on the direction of the effect).

Pairwise meta-analysis was applied to the differences between cases (patients with schizophrenia) and controls. The effect size was expressed as the bias-corrected standardized mean score (Hedges’ g).^60^ According to Cohen’s rule-of-thumb, the effect size was interpreted as small when around 0.20; moderate when around 0.50; and large when ≥0.80.^61^ When a study included more than one measure for the same outcome, all relevant measures’ effect sizes were aggregated in a single score considering the measures correlations. If this information was not reported, a default correlation between measures was set at 0.5 and dependent effect sizes were aggregated.^62^

For studies that did not report minimal information for calculating effect size, for example, they did not report measures of variations in the groups of interest, we derived the effect size from the reported statistics of 2-group analysis, either the *t* or the *F*, and the related sample sizes, according to the convertibility of *t* to effect size *d* or *g* and the known equivalence *F* = *t*^2^ (see details in the discussion about the function “escalc” of the package “metafor” 3.8-1).^63^

The results of both fixed-effect and random-effect models were reported. Fixed-effects models are aimed at making a conditional inference about the studies included in the meta-analysis and can provide valid inferences even under heterogeneity.^64^ The random-effects model provides an inference about the average effect in the entire population of studies from which the included studies are assumed to be a random selection.

Between studies variance and variance of the effect size parameters across the population were estimated with the τ^2^ statistics using the Empirical Bayes estimator,^64^ with Hartung-Knapp adjustment for random-effects model.^65^ We calculated the 95% CI for the heterogeneity using the Q-Profile method, to assess the extent and relevance of heterogeneity.^66^ Heterogeneity was assessed with Cochran’s Q and I^2^ statistics.^67^ Heterogeneity was deemed negligible when *I*^2^ < 30%; moderate for values between 30% and 60%; substantial for 75%–100% values.^68^ Egger’s regression test^69^ and the Begg’s test^70^ were applied when studies were 10 or more. With less than 10 studies in the meta-analysis, publication bias was evaluated by using the trim-and-fill procedure.^71,72^ The trim-and-fill method assumes that the most extreme results are not published and recalculates the effect size by the imputation of missing studies to produce a symmetrical funnel plot.

The radial plot was used to assess model adequacy.^73^ For each study, the observation of a large standardized residual (above 2, as a rule of thumb) suggests that the study does not fit the assumed model (ie, it may be an outlier). When studies were ≥ 10, to identify potential sources of heterogeneity, we used meta-regression to evaluate the impact of the following variables: year of publication; sample size; gender proportion; age; education; quality of the studies.

## Results

Across the 4 screened databases, 232 studies were located with data on PS in patients with schizophrenia; 100 studies were further assessed for eligibility, and 15^35,36,38,73–83^ were included in this systematic review (figure S1). As for the studies reporting data on PPS in patients with schizophrenia, 131 were initially located, 92 were further assessed for eligibility, and 5^37,84–87^ were included in this systematic review (figure S2).

### Characteristics of the Included Studies

For PS, studies were 7 from the United States; 4 from Europe (Belgium, Croatia, The Netherlands, Switzerland, 1 each); 2 from Israel; 1 from South Korea; 1 from India. In these 15 studies, mean sample size was 37 in cases (ranging from 14 to 114) and 37 in controls (14 to 120).

The proportion of male participants was reported in 14 studies, and was, on average, 72% (± 29%), ranging from 0% to 100%. The mean age (reported in 11 studies) was 33 years (± 5), ranging from 26 to 40. The mean education, calculated as school years and reported in 6 studies, was 12 years (± 1.5), ranging from 11 to 15.

For PPS, studies were 2 from the United States; 2 from Italy; 1 from France. In these 5 studies, mean sample size was 22 in cases (ranging from 18 to 27) and 26 in controls (18 to 36). The proportion of male participants was reported in 4 studies, and was, on average, 63% (±11%), ranging from 54% to 78%. The mean age (reported in 4 studies) was 40 years (± 7), ranging from 33 to 48. No information on education was reported in studies on PPS in patients with schizophrenia.

For PS, quality was rated fair in 5 studies, good in 9 studies and poor in 1 study (table S1). For PPS, quality was rated fair in 2 studies and good in 3 studies (table S2).

### Study-Defined Measures and Results

Study characteristics are reported in table 1 for PS and in table 2 for PPS.

**Table 1.** Studies Assessing Personal Space in Patients With Schizophrenia Spectrum Disorders

Study (Author, Year)	Location	Criteria and Instruments for Diagnosis	Assessment of Personal Space	Type of Sample	Sample Size	Gender (M/F)	Mean Age (SD)	Mean Personal Space (SD)	Key Findings: Statistically Significant Differences Derived From Comparisons of Samples
Horowitz et al., 1964	San Francisco, US	Not reported	Frontal Approach Distances	Schizophrenia Healthy Controls	19 19	19/0 19/0	Not reported	10.0 (8.3)	Patients tended to place greater distances around themselves than did controls
			Multidirectional Approach Distances vs Object	Schizophrenia Healthy Controls	10 10	0/10 0/10		225 205	
			Multidirectional Approach Distances vs Male	Schizophrenia Healthy Controls	10 10	0/10 0/10		697 400	
			Multidirectional Approach Distances vs Female	Schizophrenia Healthy Controls	10 10	0/10 0/10		616 371	
Tolor, 1970	Fairfield, US	Not reported	Interpersonal Distance assessed with a modification of Kuethe’s social schemata technique	Schizophrenia Healthy Controls	24 49	24/0 49/0	38.8 19.9	8.79 (0.67) 8.56 (1.11)	Patients consistently replaced the designs, neutral as well as social, closer together than controls.
Thornton & Gottheil, 1971	Philadelphia, US	Not reported	The number (n) of human-figure sets placed farther apart than the average rectangle replacement distance (0 < n < 5) using a modification of Kuethe’s social schemata technique	Schizophrenia Healthy Controls	32 17	32/0 17/0	33.8 33.0	2.88 1.71	Male patients, unlike male patients, did not display the schema that “people belong together.”
Boucher, 1972	Syracuse, US	Not reported	E-S seating distance following interview at “Intimate” distance (12 inches)	Schizophrenia Alcoholics	14 14	14/0 14/0	Not reported	76.43 69.50	Patients have larger body buffer zones than clinical controls.
			at “Personal” distance (39 inches)	Schizophrenia Alcoholics	14 15	14/0 15/0		56.50 44.07	For patients, “Personal” and “Social” seating distances led to greater attraction toward the interviewer than did “Intimate” seating distance.
			at “Social” distance (9 feet) according to Hall, 1966	Schizophrenia Alcoholics	14 16	14/0 16/0		58.00 49.36	
Duke & Mullens, 1973	Atlanta, US	Not reported	Comfortable Interpersonal Distance assessed using the Stop-Distance Technique	Schizophrenia	20	0/20	33.6	31.42 (15.26)	Chronic patients prefer greater interpersonal distances than controls
				Healthy Controls	20	0/20	33.4	17.10 (11.10)	
Srivastava & Mandal, 1990	Varanasi, India	DSM-III	Stop distance procedure Mean	Schizophrenia Healthy Controls	40 60	22/18 33/27	27.4 (6.2) 26.1 (7.0)	7.14 6.57	Patients demanded significantly greater proximal space than controls to interact with facial affect expressions, especially the nonaroused ones (happy, sad, neutral state).
			Stop distance procedure Happiness	Schizophrenia	40	22/18	27.4 (6.2)	6.47	
				Healthy Controls	60	33/27	26.1 (7.0)	3.71	
			Stop distance procedure Sadness	Schizophrenia	40	22/18	27.4 (6.2)	7.11	
				Healthy Controls	60	33/27	26.1 (7.0)	4.52	
			Stop distance procedure Fear	Schizophrenia	40	22/18	27.4 (6.2)	7.29	
				Healthy Controls	60	33/27	26.1 (7.0)	8.33	
			Stop distance procedure Anger	Schizophrenia	40	22/18	27.4 (6.2)	7.97	
				Healthy Controls	60	33/27	26.1 (7.0)	8.23	
			Stop distance procedure Surprise	Schizophrenia	40	22/18	27.4 (6.2)	7.49	
				Healthy Controls	60	33/27	26.1 (7.0)	6.91	
			Stop distance procedure Disgust	Schizophrenia	40	22/18	27.4 (6.2)	7.17	
				Healthy Controls	60	33/27	26.1 (7.0)	8.29	
			Stop distance procedure Neutral	Schizophrenia	40	22/18	27.4 (6.2)	6.47	
				Healthy Controls	60	33/27	26.1 (7.0)	6.02	
Nechamkin, 2003	Hadera, Israel	DSM-IV	CID scale vs family members	Schizophrenia	30	30/0	38.4 (10.2)	78.1 (74.8)	Patients display spacing profiles generally similar to those of controls, but a larger interpersonal distancing from family members and self-images.
				Healthy Controls	30	30/0	34.4 (10.0)	44.5 (35.4)	
			CID scale vs self images	Schizophrenia	30	30/0	38.4 (10.2)	111.8 (53.2)	
				Healthy Controls	30	30/0	34.4 (10.0)	55.9 (47.4)	
			CID scale vs significant others	Schizophrenia Healthy Controls	30 30	30/0 30/0	38.4 (10.2) 34.4 (10.0)	145.5 (52.1) 135.5 (77.1)	While positive syndrome was not associated with any kind of interpersonal distance, negative syndrome manifestations revealed substantial correlations with all the distances studied.
			CID scale vs threat related images	Schizophrenia	30	30/0	38.4 (10.2)	283.7 (57.7)	
				Healthy Controls	30	30/0	34.4 (10.0)	296.4 (45.9)	
			CID scale vs neutral people	Schizophrenia	30	30/0	38.4 (10.2)	293.9 (107.7)	
				Healthy Controls	30	30/0	34.4 (10.0)	296.4 (131.1)	
Deuš & Jokić-Begić, 2006	Zagreb, Croatia	ICD-10	Personal Space Front Distance assessed using the Stop-Distance Technique	Schizophrenia	114	54/60	Not Reported	141 (68.2)	Patients presented a larger personal space than controls
				Healthy Controls	120	53/67		81 (36.2)	
			Personal Space Left Distance assessed using the Stop-Distance Technique	Schizophrenia	114	54/60		116 (63.3)	
				Healthy Controls	120	53/67		70 (29.4)	Subjects in both groups maintained larger interpersonal distances when approached by a male experimenter and when approached frontally.
			Personal Space Rear Distance assessed using the Stop-Distance Technique	Schizophrenia	114	54/60		135 (84.3)	No significant difference in personal space preference was found between subjects manifesting paranoid and residual type of schizophrenia.
				Healthy Controls	120	53/67		75 (45.8)	
			Personal Space Right Distance assessed using the Stop-Distance Technique	Schizophrenia	114	54/60		115 (61.9)	
				Healthy Controls	120	53/67		68 (27.4)	
			Personal Space Surface assessed using the Stop-Distance Technique	Schizophrenia	114	54/60		114 (8.7)	
				Healthy Controls	120	53/67		120 (2.8)	
Park, 2009	Seoul, Korea	DSM-IV-TR	Virtual Social Environment Distance from happy person	Schizophrenia	30	16/14	28.7 (5.5)	164.15 (29.5)	Patients tended to stand farther away and have a larger angle of head orientation than the controls.
				Healthy Controls	30	16/14	26.3 (4.3)	135.1 (41.31)	
			Distance from neutral person	Schizophrenia	30	16/14	28.7 (5.5)	165 (29.65)	Compared with controls, patients showed smaller differences in distance according to the virtual person’s emotions.
				Healthy Controls	30	16/14	26.3 (4.3)	134.74 (45.91)	
			Distance from angry person	Schizophrenia	30	16/14	28.7 (5.5)	171.66 (32.9)	
				Healthy Controls	30	16/14	26.3 (4.3)	154.46 (49.86)	
			Angle head orientation vs happy person	Schizophrenia	30	16/14	28.7 (5.5)	7.38 (3.14)	
				Healthy Controls	30	16/14	26.3 (4.3)	4.12 (1.93)	
			Angle head orientation vs neutral person	Schizophrenia	30	16/14	28.7 (5.5)	7.07 (3.06)	
				Healthy Controls	30	16/14	26.3 (4.3)	3.67 (1.58)	
			Angle head orientation vs angry person	Schizophrenia	30	16/14	28.7 (5.5)	7.78 (3.65)	
				Healthy Controls	30	16/14	26.3 (4.3)	3.54 (1.64)	
Ponizovsky et al., 2013	Jerusalem, Israel	DSM-IV	CID scale vs family members	Schizophrenia	51	36/15	33.8 (10.5)	99.2 (57.5)	Compared with controls, patients were less distanced from neutral and threat- related stimuli. In patients, distancing from hostile and threat-related stimuli was associated with the severity of psychotic and affective symptoms. Self-distancing among patients was positively associated with the use of the social diversion coping, implying
				Healthy Controls	61	30/31	35.7 (11.3)	80.4 (48.8)	
			CID scale vs self images	Schizophrenia	51	36/15	33.8 (10.5)	103.7 (75.9)	
				Healthy Controls	61	30/31	35.7 (11.3)	59.5 (56.0)	
			CID scale vs significant others	Schizophrenia	51	36/15	33.8 (10.5)	178.0 (68.3)	
				Healthy Controls	61	30/31	35.7 (11.3)	158.8 (63.9)	
			CID scale vs threat related images	Schizophrenia	51	36/15	33.8 (10.5)	312.1 (75.9)	
				Healthy Controls	61	30/31	35.7 (11.3)	348.0 (20.9)	
			CID scale vs neutral people	Schizophrenia	51	36/15	33.8 (10.5)	219.2 (73.7)	Social support seeking.
				Healthy Controls	61	30/31	35.7 (11.3)	219.2 (76.6)	
Holt et al., 2015	Boston, US	DSM-IV	Stop distance procedure	Schizophrenia	15	Not reported	30.1 (9.1)	100	Patients presented a larger personal space than controls
				Healthy Controls	14		26.0 (6.5)	60	
de la Asuncion et al., 2015	Anversa, Belgio	DSM-IV-TR	The Comfortable Distance based Kennedy et al., 2009	Schizophrenia Healthy Controls	16 23	14/2 22/1	Not reported	72 45	For both the comfortable and the uncomfortable zone, patients approached the experimenter to a lesser extent than controls
			The Uncomfortable Distance based Kennedy et al., 2009	Schizophrenia Healthy Controls	16 23	14/2 22/1		31 20	
Schoretsanitis et al., 2016	Bern, Switzerland	DSM-IV	Minimally tolerable interpersonal distance assessed through the Stop-Distance Paradigm	Schizophrenia Healthy Controls	64 24	34/20 13/9	39.7 41.9	156.6 111	Comparing all patients with controls, emerged a trend to a group effect with increased interpersonal distance in patients. Patients with paranoid threat increased their minimum tolerated interpersonal distance by a factor of > 2 compared to all other patients. Patients with neutral affect did not differ from controls in the stop-distance paradigm.
Geraets et al., 2018	Groningen, The Netherlands	Schedules for Clinical Assessment in Neuropsychiatry or Comprehensive Assessment of Symptoms and History interview	Interpersonal Distance with no stressors assessed with VR	Schizophrenia	50	40/10	26.0 (4.6)	144.1 (11.9)	Interpersonal distance increased when social stressors were present in the environment. No difference in interpersonal distance regulation was found between the groups. No association was found between paranoid thoughts about the avatars during café visits and interpersonal distance.
				Healthy Controls	47	22/25	24.3 (4.3)	143.8 (9.7)	
			Interpersonal Distance in a crowded situation assessed with VR	Schizophrenia	50	40/10	26.0 (4.6)	147.5 (7.5)	
				Healthy Controls	47	22/25	24.3 (4.3)	146.7 (5.8)	
			Interpersonal Distance in a crowded situation + ethnic minority assessed with VR	Schizophrenia	50	40/10	26.0 (4.6)	148.0 (6.4)	
				Healthy Controls	47	22/25	24.3 (4.3)	145.4 (5.5)	
			Interpersonal Distance in a crowded situation + hostile expressions assessed with VR	Schizophrenia	50	40/10	26.0 (4.6)	148.0 (6.8)	
				Healthy Controls	47	22/25	24.3 (4.3)	144.0 (6.1)	
			Interpersonal Distance in a crowded situation + hostile expressions + ethnic minority assessed with VR	Schizophrenia	50	40/10	26.0 (4.6)	148.2 (6.5)	
				Healthy Controls	47	22/25	24.3 (4.3)	145.4 (5.1)	
Zapetis et al., 2022	Boston, US	DSM-V	The size of personal space assessed through the Stop-Distance Paradigm	Schizophrenia	33	22/11	30.0 (6.02)	78.61 (41.69)	Personal space size was significantly higher and its permeability was significantly lower in patients in the comparison with controls, and both measures were significantly correlated with social anhedonia and withdrawal in the full sample.
				Healthy Controls	36	22/14	28.9 (5.93)	52.53 (25.75)	
			The permeability of personal space assessed through the Stop-Distance Paradigm	Schizophrenia	33	22/11	30.0 (6.02)	57.74 (14.82)	
				Healthy Controls	36	22/14	28.9 (5.93)	67.12 (17.54)	

**Table 2.** Studies Assessing Peripersonal Space in Patients With Schizophrenia Spectrum Disorders

Study (Author, Year)	Location	Criteria and Instruments for Diagnosis	Assessment of Peripersonal Space	Type of Sample	Sample Size	Gender (% of M)	Mean Age (SD)	Mean PPS (SD)	Key Findings: Statistically Significant Differences Derived From Comparisons of Samples
Delevoye-Turrell, 2011	Lille, Villeneuve d’Ascq, Paris (France)	DSM IV	Approach/ Departing task (person vs object)	Patients with Schizophrenia	20	Not reported	Not reported	19,4 (2,1)	PPS judgments were significantly more inclined to error and more variable in the patients than in the controls
				Healthy controls	20			10 (2,1)	
			Approach/ Departing task (person vs person)	Patients with Schizophrenia	20			18,9 (6,7)	PPS judgments were significantly more inclined to error and more variable in the patients than in the controls
				Healthy controls	20			15,1 (4)	
Di Cosmo, 2018	Chieti (Italy), Colchester (UK)	PANSS, SPQ	Reaction Times (RTs) to tactile stimuli coupled with dynamic approaching sounds	Patients with Schizophrenia	18	66,6	34,7 (8,2)	380 (25)	Patients show significantly narrower PPS boundary
				Healthy controls	18	66,6	35 (9,1)	308 (14)	
			Reaction Times (RTs) to tactile stimuli coupled with dynamic approaching sounds	Low Schizotypy Individuals	20	66,6	34,7 (8,2)	274 (10) 309 (12)	High-schizotypy individuals show a significantly narrower PPS boundary
				High Schizotypy Individuals	24	66,6	35 (9,1)		
Noel, 2020	Nashville (USA)	DSM IV SCID IV	Reaction Times (RTs) to tactile stimuli coupled with congruent or incongruent visual stimuli, RTs as a function of PPS size	Patients with Schizophrenia	18	55,09	45,09 (9,94)	aOR = 0.18, CI95[0.04, 0.74], p = .0175	Schizophrenia as a diagnostic group was not a significant predictor of PPS size
				Healthy controls	33	63,89	33,56 (11,19)		
			Reaction Times (RTs) to tactile stimuli coupled with congruent or incongruent visual stimuli, RTs as a function of slope of the gradient of PPS boundary	Patients with Schizophrenia	18	55,09	45,09 (9,94)	aOR = 1.4, CI95[0.35, 5.67], p = .6344	A diagnosis of SZ did not hold significant predictive power as a determinant of PPS gradient
				Healthy controls	33	63,89	33,56 (11,19)		
Lee, 2021	Nashville (USA)	DSM V	Visuotactile reaction time procedure in virtual reality in a social condition	Patients with Schizophrenia	24	54,16	48,82 (9,24)	1,04 (0.16)	The PPS size was significantly smaller in individuals with SZ in the social condition
				Healthy controls	24	45,83	47,96 (9,38)	1,15 (0,15)	
			Visuotactile reaction time procedure in virtual reality in a non-social condition	Patients with Schizophrenia	24	54,16	48,82 (9,24)	Not reported	There was no significant diference in PPS size between patients and controls non social condition
				Healthy controls	24	45,83	47,96 (9,38)		
Ferroni, 2022	Parma, Chieti (Italy), Paris (France)	DSM V	Peripersonal space task Reaction Time before a motor training	Patients with Schizophrenia	27	77,7	32,55 (2,45)	1455,03 (SE: 36,76)	A narrower PPS extent was found in SCZ than in healthy controls
				Healthy controls	32	43,7	28 (2,73)	1306,08 (SE: 33,76)	
			Peripersonal space task, Reaction Time after a motor training	Patients with Schizophrenia	27	77,7	32,55 (2,45)	1431,38 (34,74)	After tool-use, the PPS is larger in HC than in SCZ but both groups show an equal PPS expansion
				Healthy controls	32	43,7	28 (2,73)	1329.73 (35.81)	
			Peripersonal space task Difference Limen values before a motor training	Patients with Schizophrenia	27	77,7	32,55 (2,45)	696,43 (30,88)	Only SCZ show steeper PPS boundaries after a motor training
				Healthy controls	32	43,7	28 (2,73)	504,41 (28,37)	
			Peripersonal space task Difference Limen values before a motor training	Patients with Schizophrenia	27	77,7	32,55 (2,45)	613,28 (28.76)	Only SCZ show steeper PPS boundaries after a motor training
				Healthy controls	32	43,7	28 (2,73)	Not reported	

The 15 studies assessing PS used several methodologies, most of all estimating its length; 6 studies used the Stop Distance paradigm (Box 1). One study reported no differences in PS between patients and controls,^88^ while all other studies found that the PS was larger in patients with schizophrenia than in controls. Among the 5 studies assessing PPS, 3 studies had relatively homogeneous metrics of the measures (reaction time), and the remaining 2 had rather heterogeneous measures. Irrespective of the measure, 1 study found no differences in PPS between patients and controls,^85^ whereas 4 others found a narrower PPS in patients than in controls.

### Meta-analysis of Studies on Personal Space in Patients With Schizophrenia

Twelve studies were included in the meta-analysis. Three studies^75,76,78^ were excluded since they did not report enough information to calculate an effect size. All studies included in the meta-analysis compared patients with schizophrenia to healthy controls.

**Fig. 1.**
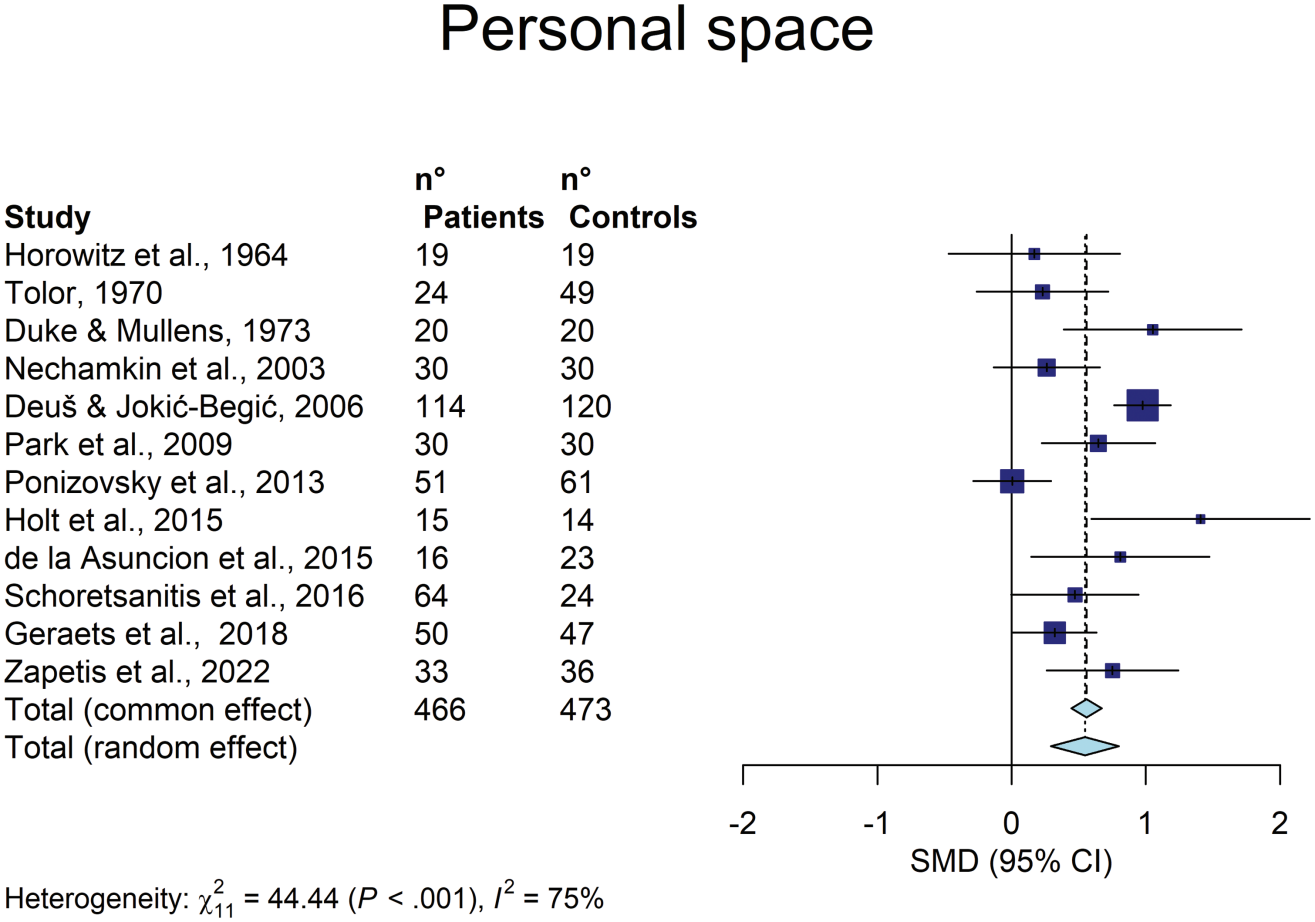
Forest plot of the effect sizes of the personal space differences, calculated as Hedges’ g, in the comparison between patients with schizophrenia and controls.

Out of 12 samples, the results included 466 patients with schizophrenia and 473 controls. Overall, patients put a larger interpersonal distance from the stimuli than controls with a moderate effect size in both the fixed effect (Hedges’ g = 0.558 [95%CI: .445–.671]; *z* = 9.67; *P* < .0001) and the random effects model (0.547 [0.294–0.799]; *z* = 4.77; *P* = .0006) (**figure 1**).

Heterogeneity was substantial in this meta-analysis: Cochran’s Q = 44.44; df = 11; *P* < .0001; *I*^2^ = 75%% (95% CI: 56%–86%). This is likely a reflection of differences among the samples in terms of measure of PS, duration of illness, type of the disorder (with or without paranoid delusions), and stage of the condition (first-episode vs recurrence or chronicity of the episode).

The funnel plot was reasonably symmetric (figure S3), with no evidence of publication bias at the Egger’s or the Begg’s test.

Studies using the Stop Distance paradigm resulted in a larger interpersonal distance from the stimuli than controls in both the fixed-effect (Hedges’ g = 0.908 [0.739–1.078] vs 0.276 [0.124–0.428]; between groups Q = 29.69; df = 1; *P*≤ .0001) and the random-effects model (0.883 [0.529–1.236] vs 0.300 [0.060; 0.540]; between groups Q = 13.12; df = 1; *P* = .0003) (figure S4). Heterogeneity accounted for by differences in measurement was substantial (77%), with residual heterogeneity not statistically significant (Q = 14.74; df = 10; *P* = .142). Larger PS was seen in samples with a greater proportion of women (figure S5), with estimates negatively related to the proportion of men in the sample (beta = −0.009; SE = 0.003; t = −2.810; *P* = .020). The heterogeneity accounted for by the effect of gender proportion was 59%. Essentially, about half of the heterogeneity in the estimates depended on the gender proportion in the sample. It should be noted that studies applying the Stop-Distance paradigm included a lower proportion of men than those using different methods to estimate PS (on average, 42% ± 29% vs 85% ± 18%).

There was no further effect on the estimates of age (beta = −0.023; SE = 0.028; *t* = −0.822; *P* = .438); education (beta = 0.287; SE = 0.092; *t* = 3.107; *P* = .089); the quality of the studies (*F*[1;10] = 0.294; *P* = .599); sample size (beta = 0.001; SE = 0.002; *t* = 0.361; *P* = .725); or the year of publication (beta = 0.003; SE = 0.006; *t* = 0.494; *P* = .632).

Finally, the radial plot indicated a good fit of the (random effects) model, with no evidence of influential points or outliers affecting the estimates (figure S6).

### Meta-analysis of Studies on PPS in Patients With Schizophrenia

All 5 studies were included in the meta-analysis, totaling 113 patients with schizophrenia and 130 controls. Overall, patients showed a narrower PPS than the controls at the fixed effect (Hedges’ g = 1.043 [95%CI: .739–1.348]; *z* = 6.72; *P* < .0001) but not at the random effects model (1.318 [−0.721 to 3.359]; *z* = 1.79; *P* = .147).

**Fig. 2.**
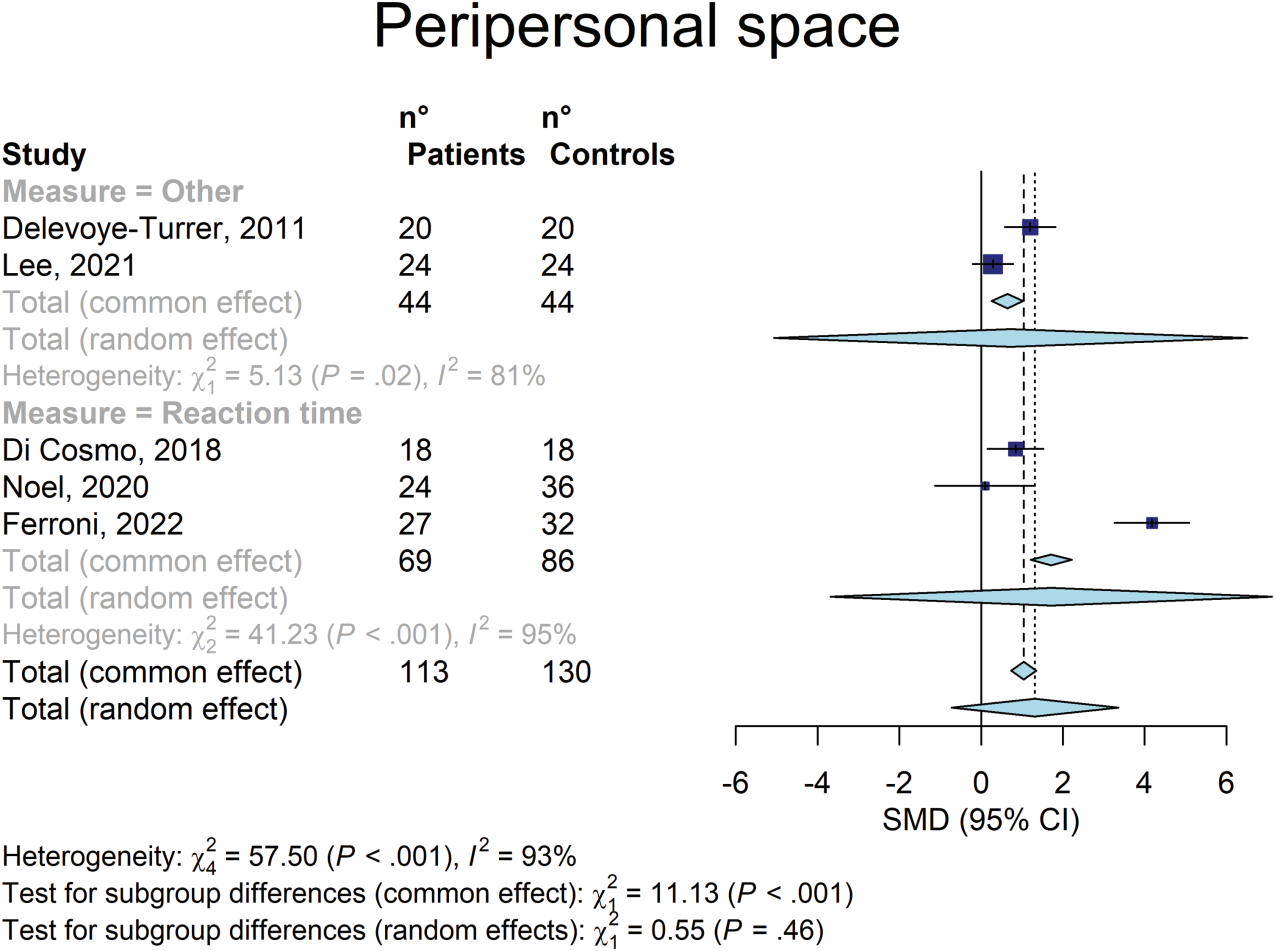
Forest plot of the effect sizes of the peri-personal space differences, calculated as Hedges’ g, in the comparison between patients with schizophrenia and controls.

A lack of statistical significance in the random effects model was observed even when the studies with inhomogeneous measures were evaluated separately (**figure 2**).

Heterogeneity was substantial in this meta-analysis: Cochran’s Q = 57.50; df = 4; *P* < .0001; *I*^2^ = 93% (95% CI: 87%–96%).

The funnel plot and Egger’s and Begg’s test were not evaluated since the studies were less than 10. However, the trim-and-fill method did not suggest additional studies to be added to correct for publication bias. No meta-regression was applied since studies were less than 10.

The radial plot indicated a good fit of the (random effects) model, with no evidence of influential points or outliers affecting the estimates (figure S7).

## Discussion

This is the first systematic review and meta-analysis addressing modifications of PS and PPS body-centric metrics in schizophrenia. PS is a subjective and culturally influenced concept related to an individual’s comfort zone for interpersonal interactions, whereas PPS is a neuroscientific concept related to the region of space around the body that is actively monitored to facilitate physical actions and interactions with the environment. The results indicate that PS and PPS are differently affected in patients with schizophrenia in comparison with controls: the PS is consistently enlarged and the PPS is narrower.

There is an effect of gender, with women showing a preference for a larger interpersonal distance than men. We cannot exclude that the larger PS found in Stop-Distance studies might be influenced by the percentage of female participants with a male confederate, since women might prefer more space from male confederates.^40,41^ Such effect seems to be in agreement with greater amygdala activation in response to violations of PS in healthy women compared with men.^89^ The estimated effect size was in the moderate range and was robust enough to be found in both the fixed and random-effects model. In the current set of studies, 9 studies out of 15 had a sample size of 30 participants or more. However, efforts to enroll larger sample sizes are necessary to confirm and expand the current findings in the field.

Although we found no indications of publication bias, heterogeneity was substantial and, albeit reduced to half by gender effect, remained elevated because of unmeasured factors. Age, education, sample size, publication year, and quality of the studies did not impact the estimates nor reduced heterogeneity. The wide variability in the methods used to measure PS as well as in the metric applied in the included studies contributed to the heterogeneity, yet they were too disparate to be used for sensitivity analysis.

The studies on PS in schizophrenia had variable quality, but most reached a good level rating. Moreover, quality did not impact estimates in the meta-analysis. Nevertheless, 3 out of 15 studies included in the systematic review did not report enough information to calculate an effect size and could not be included in the meta-analysis. The reporting of observational studies in the field has improved, but essential information (eg, variance) continues to be missing in studies published after 2015.

With respect to PPS, the meta-analytical results indicate that PPS in schizophrenia is narrower at the fixed effect model but not at the random-effects model. The estimated effect size was large and at the lower bound estimate in the fixed effect model (0.74). The trim-and-fill method suggested no publication bias, but heterogeneity was substantial. The result cannot be extended to the population from which the studies were supposedly extracted (based on the random-effects model) and, at most, can be considered preliminary, pending further exploration of the topic.

### Strengths and Limitations

We thoroughly reviewed all available literature on the topic and applied state-of-the-art statistics to analyze the extracted estimates. However, several limitations, mostly intrinsic to the primary studies, have to be considered. First, due to the relatively low number of included studies, particularly for PPS, the findings should be considered exploratory. Overall, there was limited data from the papers which did not allow further multivariate analysis, and, when reported, symptomatic correlates were too heterogeneous to allow the application of meta-regression techniques. Moreover, studies on PPS are too few to derive solid estimates, and we could not perform sensitivity analyses to investigate the source of the retrieved heterogeneity. Nevertheless, the quantitative synthesis of even a few studies is preferable to their narrative review.^90^ Across the studies, there was a noticeable variability in the methods applied to measure PS or PPS. Therefore, we cannot rule out that the differences in PS and PPS detected in this meta-analysis might also be influenced by variations in their empirical assessment.

### Implications: For the Research and for the Clinics

Despite intrinsic limitations of current primary literature on PS and PPS, this systematic review and meta-analysis found preliminary evidence of an enlarged PS and a narrowed PPS in patients with schizophrenia, thereby supporting the partial distinction of these body-centric spatial constructs as they were conceptualized and measured up to now. Such pattern coheres with the dynamic relationship between PS and PPS and their different putative functions. PS, indeed, circumscribes a sphere of perceived intimacy not to be intruded by others, whereas PPS defines a sphere of potential action of proactive interactions, capturing a space of enactive possibilities.

If the borders of PPS represent the extreme of the transition area from the active Self to the world, the narrowing of the PPS in schizophrenia would be subjectively felt as a disturbingly excessive proximity of the surrounding world. This would relate to an extension of the PS so that the person would need a larger space to feel safe or at least not intruded by others.

The preliminary finding of a basic pattern of altered body-centric spatial metrics in schizophrenia (ie, narrower PPS and larger PS), emerging from the current meta-analysis, invites further speculative hypotheses concerning its ontogenesis and its relationship to the lived experience of space (*espace vécu*) and to super-ordinate psychotic experiences (such as paranoid threats, passivity and autocentric-like experiences).

Is the subjective feeling of being overexposed to others somehow rooted in the subjective metrics of how the interpersonal distance is processed? Do autocentric-like experiences and paranoid thoughts influence how PS/PPS is subjectively and implicitly processed? Do altered metrics of the PS–PPS and altered felt Self-Other boundaries represent different explanatory facets or levels of description (ie, neurocognitive and phenomenological) of the same or converging phenomena?

Current empirical literature is clearly insufficient to solve this puzzle; however, further studies contextually assessing PS and PPS in individuals at different stages of schizophrenia and its spectrum conditions (eg, schizotypal personality vs clinical high-risk for psychosis vs first episode psychosis vs schizophrenia) would be a crucial step forward. Nonetheless, keeping in due consideration, the ontogenesis of body-centered spatial metrics and the deviations that may lead to the pattern emerging at a meta-analytical level in schizophrenia (larger PS, narrower PPS), could deepen our understanding of the developmental features of vulnerability to schizophrenia.

First, the bodily Self and its surrounding zone is processed by a multimodal sensory integration. Such integration is altered in schizophrenia from early premorbid stages, as detected in subjects presenting so-called schizotaxic vulnerability (eg, offspring of schizophrenic patients).^91^ A potential involvement of impaired corollary discharges may be implied in the disrupted multimodal sensory integration that, over development, could interfere with the formation of a nuanced implicit connection with the bodily Self, encompassing aspects of ownership and agency.^25–27^ This, over time, could contribute to the altered embodiment phenomenologically manifested in basic Self-disorders.^31,32^ Consequently, a neuro-developmental perspective on the emergence and shaping of PS and PPS along the trajectory leading to schizophrenia should account for this constraint associated with a deficit in multimodal sensory integration.

## Conclusions

Lived space (aka spatiality) encompasses the way we feel the surrounding space and is inseparable from our immersion in the world as embodied, active subjects. Therefore, spatiality, as a basic experiential background, is largely a preverbal and, although we do not ordinarily reflect on it, fully permeates and affects the way we feel. This systematic review and meta-analysis found evidence of an enlargement of PS and a contraction of PPS in individuals with a diagnosis of schizophrenia in comparison with nonaffected controls. The need of a larger safe area and the reduced area of active interaction with the surrounding are in line with the modifications of the lived space (*espace vécu*) which thematized by phenomenological psychopathology, and which exhibit a paroxysmic amplification in many psychotic states, for example, aberrant salience and intrusiveness of surrounding meanings, passivity experiences, and persecutory delusions.

Understanding the ontogenetic emergence and development of PS and PPS in individuals diagnosed with schizophrenia poses a challenge in establishing a clear causal relationship between developmental, neurocognitive, and phenomenological levels of description. Nonetheless, it seems plausible that the ontogenesis of PS and PPS could be an important subcomponent of the neurodevelopmental model of schizophrenia worth addressing. Furthermore, within the ontogenetic processes of PS/PPS formation, early alterations in multimodal sensory integration presumably play a crucial role, influencing the perception of one’s bodily Self and its surrounding space, including the primary relationship with the caregivers.

## Supplementary Material

Supplementary material is available at https://academic.oup.com/schizophreniabulletin/.

sbae159_suppl_Supplementary_Figures_S1-S7_Tables_S1-S2
