# Supplementary material for: L’espace vécu and Its Perturbations in Schizophrenia: Systematic Review and Meta-analysis of Altered Body-Centric Metrics—Personal and Peripersonal Space
Source: Schizophr Bull. 2024 Oct 3;51(3):578–94. doi: 10.1093/schbul/sbae159 (PMC12061664; doi:10.1093/schbul/sbae159)
Supplement: sbae159_suppl_Supplementary_Figures_S1-S7_Tables_S1-S2 [file sbae159_suppl_supplementary_figures_s1-s7_tables_s1-s2.docx]

**Supplementary materials**


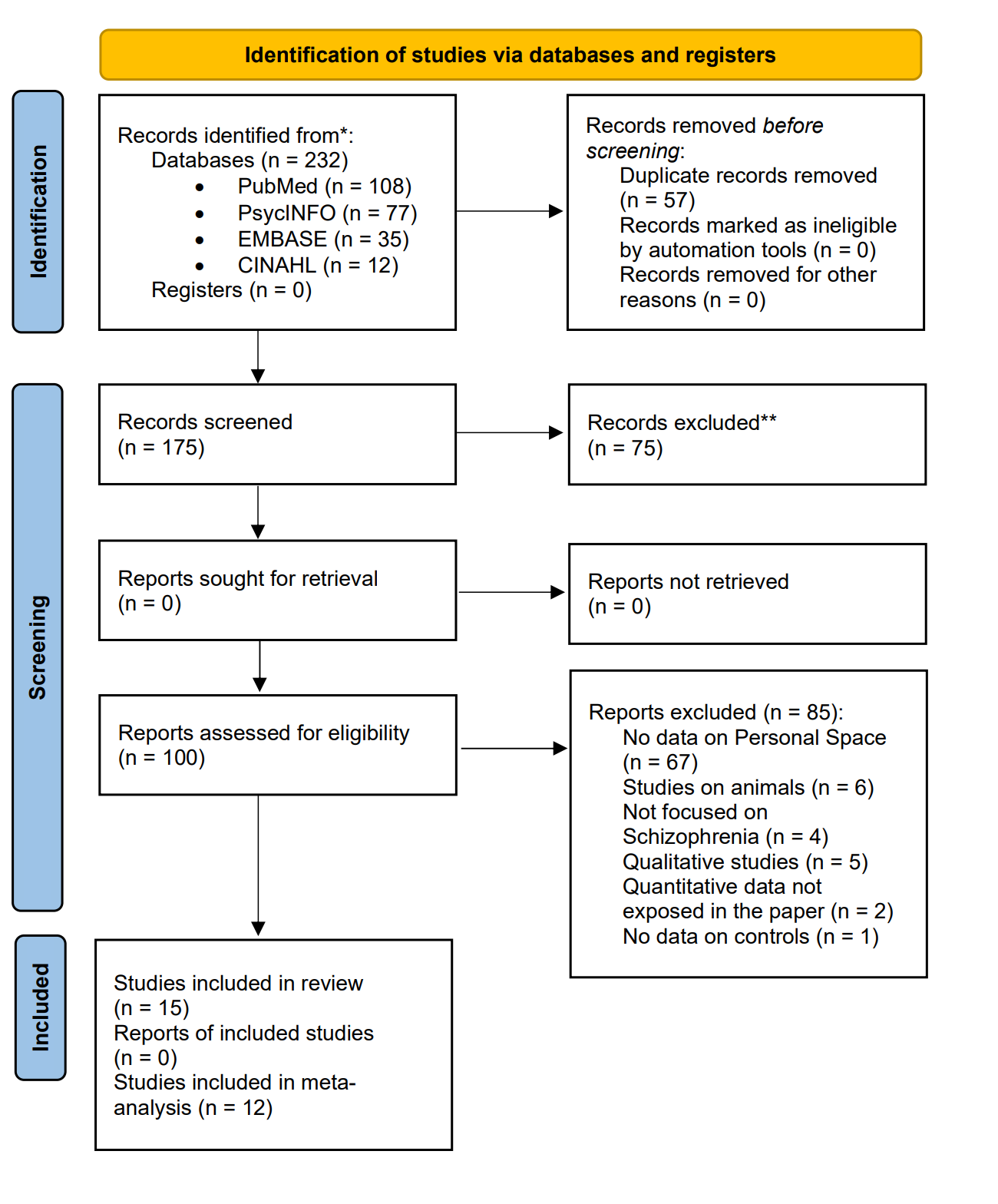


Figure S1. PRISMA flowchart of studies reporting data on personal space in cases (patients with schizophrenia) and controls.


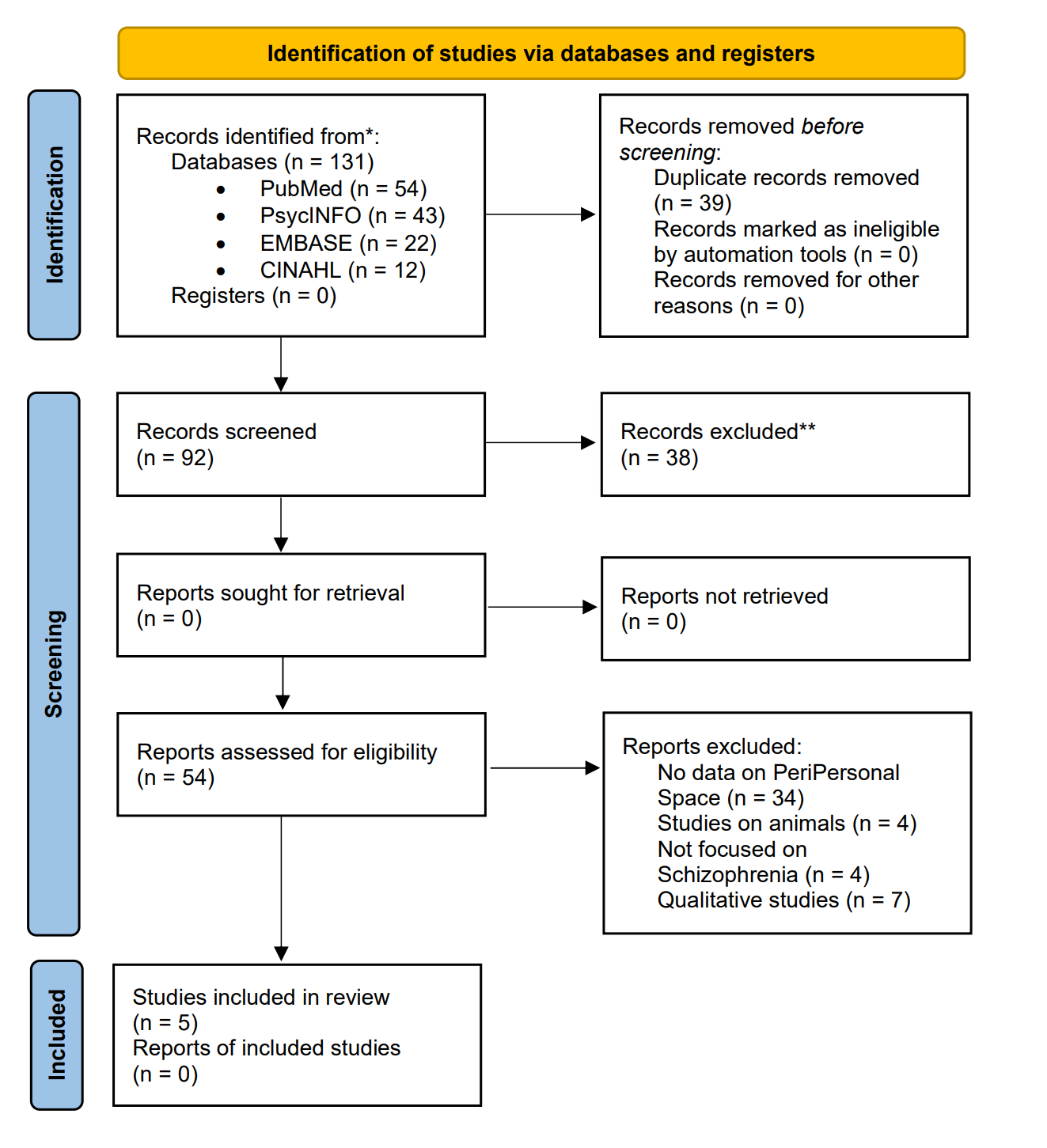


Figure S2. PRISMA flowchart of studies reporting data on peri-personal space in cases (patients with schizophrenia) and controls.


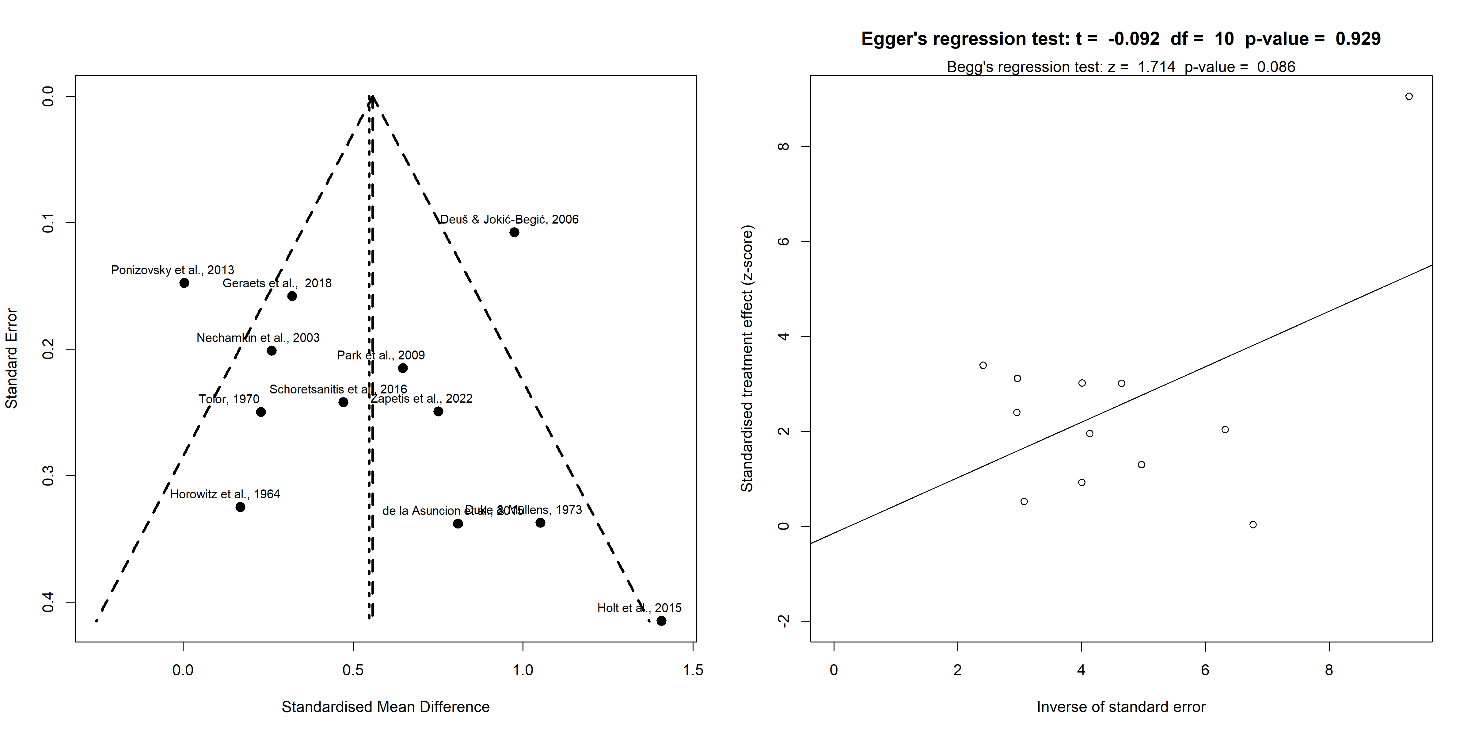


Figure S3. Funnel plot (on the left) and the results of the Egger’s and the Begg’s test for the global meta-analysis of the difference in personal space between cases (patients with schizophrenia) and controls.


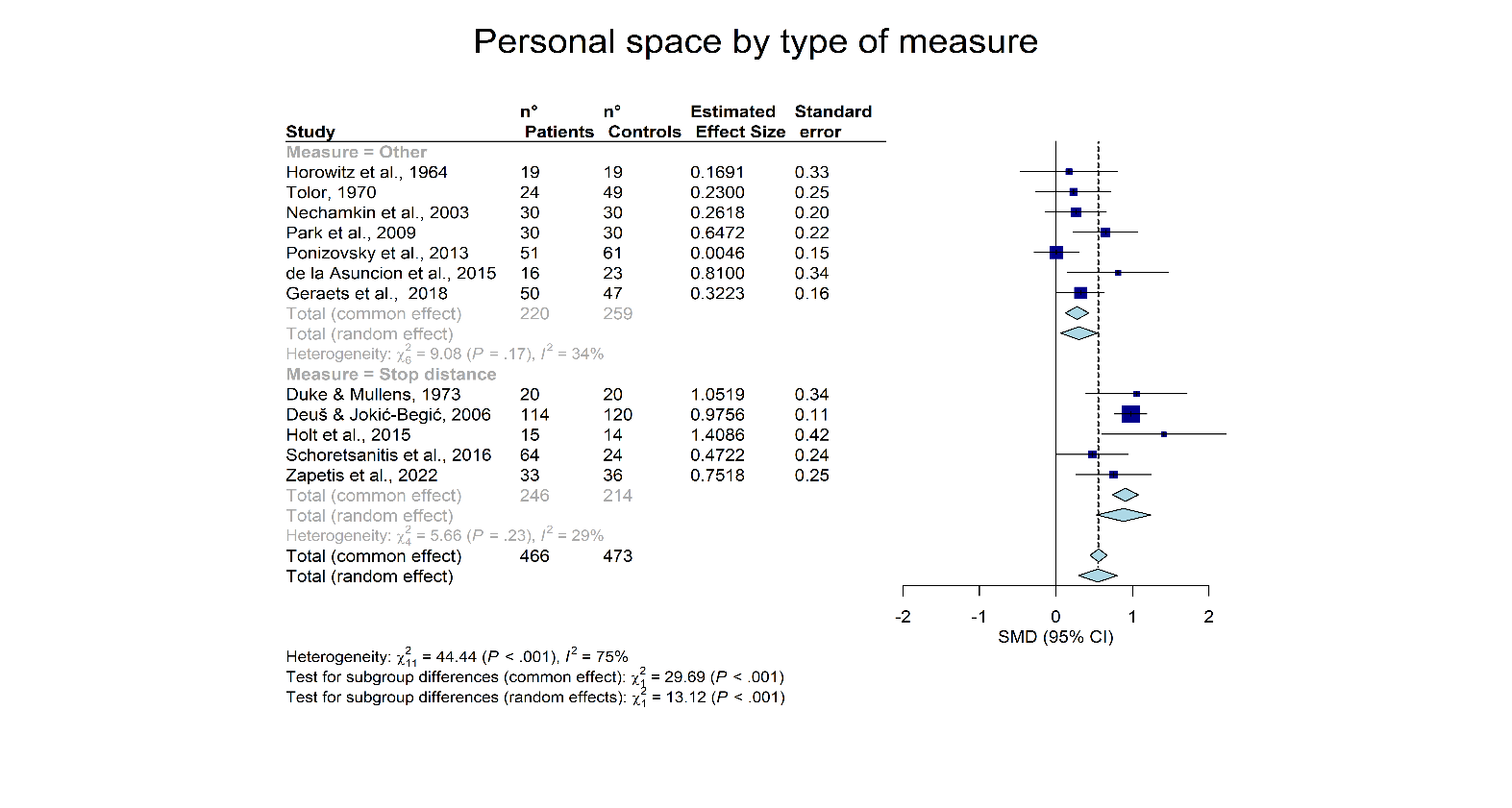


Figure S4 Forest plot of the effect sizes of the personal space differences, calculated as Hedges’ g, in the comparison between patients with schizophrenia and controls, according to type of measure


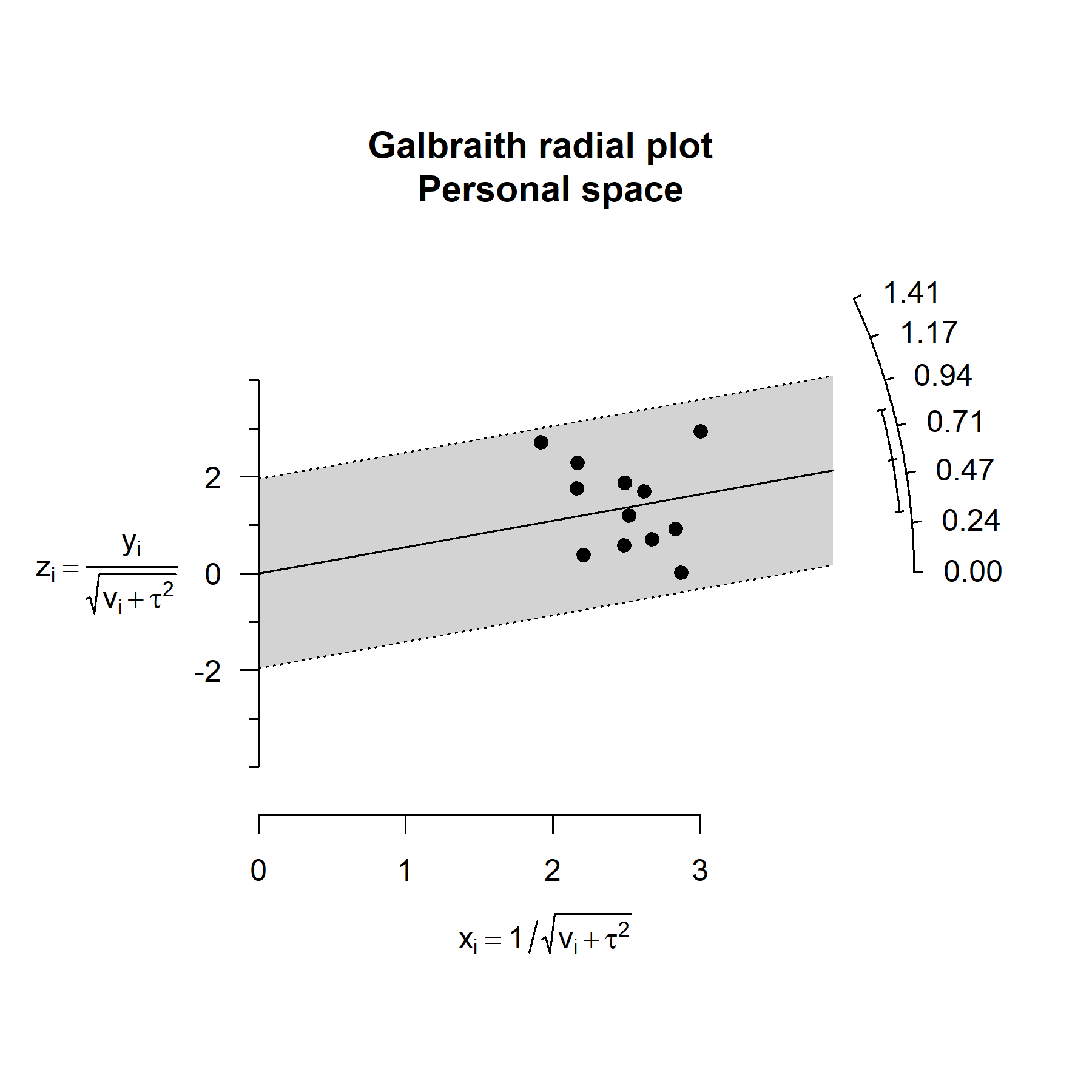


Figure S5. (Galbraith) Radial plot of the standardized effect (in z-scores, on the vertical axis) against the inverse of standard error (on the horizontal axis) in studies comparing personal space between patients with schizophrenia and controls.


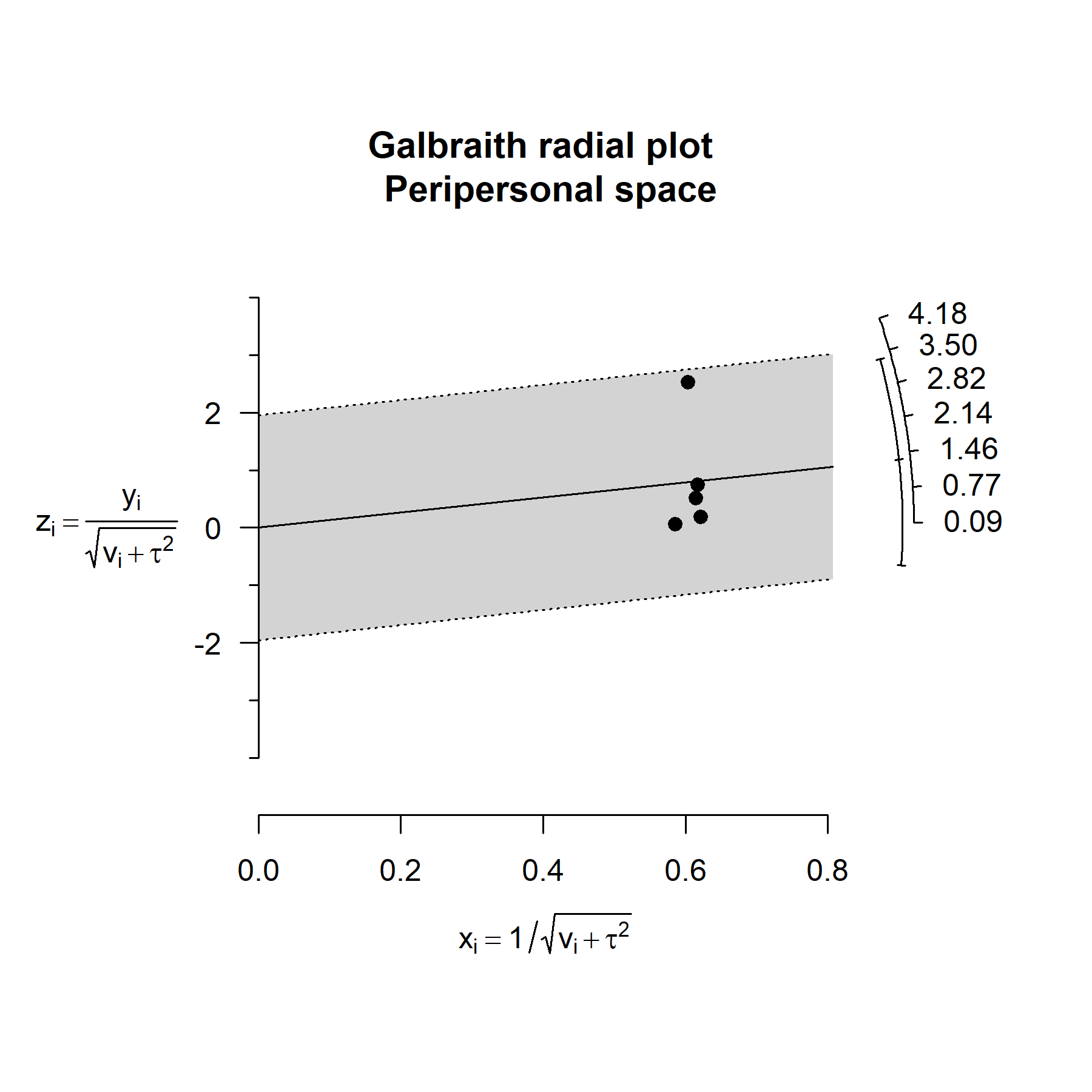


Figure S6. (Galbraith) Radial plot of the standardized effect (in z-scores, on the vertical axis) against the inverse of standard error (on the horizontal axis) in studies comparing peri-personal space between patients with schizophrenia and controls.

**Table S1** Quality rating of studies included in the Meta-analysis according to the Newcastle-Ottawa Assessment Form for Case control studies on personal space

| **Study** | **Selection** | | | | **Comparability** | **Outcome** | | | **AHRQ**  **Standard** |
| --- | --- | --- | --- | --- | --- | --- | --- | --- | --- |
|  | **Item 1** | **Item 2** | **Item 3** | **Item 4** | **Item 1** | **Item 1** | **Item 2** | **Item 3** |  |
| Horowitz et al., 1964 |  | X | X |  | X |  | X | X | Fair |
| Tolor, 1970 |  | X | X |  | X |  | X | X | Fair |
| Thornton & Gottheil, 1971 |  | X | X |  | X |  | X | X | Fair |
| Boucher, 1972 |  | X |  |  | X |  | X | X | Poor |
| Duke & Mullens, 1973 |  | X | X |  | X |  | X | X | Fair |
| Srivastava & Mandal, 1990 | X | X | X |  | X |  | X | X | Good |
| Nechamkin, 2003 | X | X | X |  | X |  | X | X | Good |
| Deuš & Jokić-Begić, 2006 |  | X | X |  | X |  | X | X | Fair |
| Park, 2009 | X | X | X |  | X |  | X | X | Good |
| Ponizovsky et al., 2013 | X | X | X |  | X |  | X | X | Good |
| Holt et al., 2015 | X | X | X |  | X |  | X | X | Good |
| de la Asuncion et al., 2015 | X | X | X |  | X |  | X | X | Good |
| Schoretsanitis et al., 2016 | X | X | X |  | X |  | X | X | Good |
| Geraets et al., 2018 | X | X | X |  | X |  | X | X | Good |
| Zapetis et al., 2022 | X | X | X |  | X |  | X | X | Good |

*Note: Newcastle-Ottawa Quality Assessment Form for Case control studies – Selection items*

*1) Case definition*

*2) Representativeness of the cases*

*3) Selection of controls*

*4) Definition of controls*

*Comparability Items*

*1) Comparability of cases and controls on the basis of the design or analysis controlled for confounders*

*Exposure items*

*1) Ascertainment of exposure*

*2) Same method of ascertainment for cases and controls:*

*3) Non response rate*

*Thresholds for converting the Newcastle-Ottawa scales to AHRQ standards (good, fair, and poor):*

*Good quality: 3 or 4 stars in selection domain AND 1 or 2 stars in comparability domain AND 2 or 3 stars in exposure domain*

*Fair quality: 2 stars in selection domain AND 1 or 2 stars in comparability domain AND 2 or 3 stars in exposure domain*

*Poor quality: 0 or 1 star in selection domain OR 0 stars in comparability domain OR 0 or 1 stars in exposure domain*

**Table S2** Quality rating of studies included in the Meta-analysis according to the Newcastle-Ottawa Assessment Form for Case control studies on peripersonal space

| **Study** | **Selection** | | | | **Comparability** | **Outcome** | | | **AHRQ**  **Standard** |
| --- | --- | --- | --- | --- | --- | --- | --- | --- | --- |
|  | **Item 1** | **Item 2** | **Item 3** | **Item 4** | **Item 1** | **Item 1** | **Item 2** | **Item 3** |  |
| Delevoye-Turrell et al. 2011 | X |  | X |  | X |  | X | X | Fair |
| Di Cosmo et al. 2018 | X | X |  |  | X |  | X | X | Fair |
| Noel et al. 2020 | X | X | X |  | X |  | X | X | Good |
| Lee et al. 2021 | X | X | X |  | X |  | X | X | Good |
| Ferroni et al. 2022 | X | X | X |  | X |  | X | X | Good |

*Note: Newcastle-Ottawa Quality Assessment Form for Case control studies – Selection items*

*1) Case definition*

*2) Representativeness of the cases*

*3) Selection of controls*

*4) Definition of controls*

*Comparability Items*

*1) Comparability of cases and controls on the basis of the design or analysis controlled for confounders*

*Exposure items*

*1) Ascertainment of exposure*

*2) Same method of ascertainment for cases and controls:*

*3) Non response rate*

*Thresholds for converting the Newcastle-Ottawa scales to AHRQ standards (good, fair, and poor):*

*Good quality: 3 or 4 stars in selection domain AND 1 or 2 stars in comparability domain AND 2 or 3 stars in exposure domain*

*Fair quality: 2 stars in selection domain AND 1 or 2 stars in comparability domain AND 2 or 3 stars in exposure domain*

*Poor quality: 0 or 1 star in selection domain OR 0 stars in comparability domain OR 0 or 1 stars in exposure domain*
